# Decision in the Celluloid Case

**Published:** 1877-12

**Authors:** 


### EDITORIAL, ETC.

---

We are indebted to Albert Ritchie, Esq., for the following decision:

*Circuit Court of the United States, District of Massachusetts. In Equity. No. 729a—Goodyear Dental Vulcanite Company vs. Charles G. Davis. Same vs. Defendants in sixty-seven other cases.*

Opinion of the Court.—Shepley, J.

The patent and its reissues, granted for the invention of John A. Cummings, of "an improvement in artificial gums and palates," or, as described in the claim, "the plate of hard rubber, or its equivalent, for holding artificial teeth, or teeth or gums substantially as described," have been the subject of extensive and prolonged litigation. Since the affirmance by the Supreme Court of the United States in *Smith Appellant*, vs. *Goodyear Dental Vulcanite Campany*, 3 Otto, 486, of the degree of the Circuit Court in the test case of *Goodyear Dental Vulcanite Company* vs. *Smith*, the validity of the reissued patent has been fully established. The decision of the Supreme Court in that case must be considered as final, not only as to the validity but as to the construction of the patent, which was carefully considered in that case, both in the Circuit and the Appellate Court, as it had previously been in the Wetherbee and the Gardiner cases.

In *Vulcanite Company* vs. *Smith*, in the First Circuit, this Court decided that the patent was not for a process or art, but for the new product resulting from the manipulation by the described new process, and for one of those products in which the process so inheres that the described product can only be made by the described process, and that the invention was one in which the process by which it is made is a part of the substance, the thing made, the manufacture. What the new product was, and wherein the novelty consisted in the process, we shall hereafter have occasion to consider. In the opinion (by Mr. Justice Strong) in the Supreme Court in the same case, the invention patented is thus described : " The invention, then, is a product or manufacture made in a defined manner. *It is not a product alone separated from the process by which it is created.* The claim refers in terms to the antecedent description without which it cannot be understood. The process detailed is thereby made as much a part of the invention as are the materials of which the product is composed."

If the defendants, by practising the process described by Cummings, using the materials described by him, or such materials as are equivalents, and were known equivalents at the date of his invention, in the described process, or such as, in the process, are mere substitutes of one material for another, without any change in the process or in the effect, have produced a product the equivalent of his in the described properties, and for the described functions, then, and only then, have they infringed.

The product, the new article of manufacture patented, was a set of artificial teeth, consisting of a plate of hard rubber or vulcanite, with teeth or teeth and gums secured thereto in the manner described in the patent, by imbedding the teeth and pins in the vulcanizable compound, so that it shall surround the teeth and pins while the compound is in a soft state before it is vulcanized, so that, when the compound is vulcanized, the teeth are firmly secured by the pins imbedded in the vulcanite, and there is a tight joint between the vulcanite and the teeth. This product was a new product, not alone because it substituted one material for another, the material, vulcanite, rigid enough for purposes of mastication, yet pliable enough to yield a little to the mouth, and at the same time light and inexpensive, in place of the hard, uny elding, expensive and heavy metals previously used, but also in the additional fact that it was made by a process which, taken as a whole, was a new process, by the use of which process " the teeth can easily be baked into the gums, which form one piece with the plate."

The next question to be considered is, what was there new in the described process. The whole process of forming and making a set or case of teeth, including the plate, gums and teeth is fully described in the Cummings patent, and this process is fully described in the case of *Vulcanite Company* vs. *Smith*, that it becomes unnecessary to repeat it here. It is sufficient to state that in view of the state of the art there was nothing substantially new in that process until we reach this part of the description: "The teeth are provided with pins projecting, "therefrom in such manner that the rubber which is to constitute the plate will close around them, and by means of them "hold or secure the teeth permanently in position. The plaster "mould, with the teeth adhering therein, as just described, is "now filled with soft rubber, a little at a time, pressed in with "the finger, or in any other convenient way; and care is to be "taken that the rubber is made to completely fit into the cav- "ities, and around the protuberances, including the pins, and "is filled in to the thickness or depth desired to form the plate. "I then lock the rubber plate in position by shutting the other "half of the plaster mould over it, to insure its retaining its "exact form while warming, and then heat or bake it in an "oven, or in any other suitable way. The soft rubber or gum, "so inserted in the mould, is to be compounded with sulphur, "rubber, etc., in the manner prescribed in the patent of Nelson "Goodyear, dated May 6, A. D. 1851, for making hard rubber, "and is to be subjected to sufficient heat to vulcanize or harden "it substantially, as directed in that patent. It is also to be "colored in imitatiou of the natural gums, by mixing it with "vermillion, or other suitable coloring matter, while in the soft "state. After the plate has been heated sufficiently to harden "it or convert it into hard rubber or 'vulcanite,' so called, the "mould is removed, and the plate is polished ready for use."

It will thus be seen that an essential element of the described product, is "a plate of hard rubber or vulcanite," in which the teeth are imbedded, and an essential ingredient in the described process, is the soft rubber or gum, compounded with sulphur, rubber, etc., in the manner prescribed in the patent of Nelson Goodyear, for making hard rubber, and that an essential step in the described process is the subjecting the compound of soft rubber or gum with sulphur "to sufficient heat to vulcanize or harden it, substantially as described in that patent," (*i. e.* the patent of Nelson Goodyear, of May 6, A. D. 1851.)

The equivalent of that product thus made by that process must therefore contain the equivalent of the plate of hard rubber made and manipulated by a process equivalent to the described process of compounding a gum with sulphur, and applying it and moulding it and incorporating it with the teeth and gums, when in a soft state and then subjecting it to heat, to harden and vulcanize it in the manner described in the Goodyear patent or in some equivalent manner, or by some equivalent process.

The defendants use, in making their set of artificial teeth, a plate made of "celluloid," substantially a new material, discovered and patented since the date of the Cummings invention. This substance is compounded of cellulose or vegetable fibre and camphor. No rubber or other equivalent gum, and no sulphur or equivalent for sulphur in the process, enter into its ingredients. It is not a vulcanizable compound, and contains no vulcanizing agents, in its composition. The camphor in its composition, instead of being a vulcanizing agent, causes the composition to soften instead of harden under the influence of heat. The product, when compounded, and before being subjected to heat, is not soft, like soft rubber under like conditions but hard. In the manipulation of this material, the process of making a set of teeth, composed of the plate and teeth and gums, is an entirely different process from the process described in the Cummings patent, when compared with that part of the Cummings process which was new in the state of art, and the novelty of which part gave to the Cummings process, when considered as a whole, the ingredient of novelty and patentab lity. It is not placed in the mould in a soft, plastic condition, " a little at a time, pressed in with the finger, or in any other convenient way," but in a hard, rigid condition, like horn or bone or ivory. It is then subjected to heat, not to vulcanize or harden, but to soften it. It afterwards, on being cooled or restored to its original temperature, returns to its original condition as a hard substance, as when first placed in the mould. No vulcanizing process, or even process of hardning by heat, and no equivalent for any such process, is practised. In the light of these comparisons, it appears evident to the Court that the use of celluloid in the manufacture of sets of artificial teeth, as practised by the defendants, and the manufacture itself, the set or plate of teeth, differ as much, both as to process and product, from the process and product described and claimed in the Cummings patent, as that process and that product differed from those previous manufactures which existed before the Cummings invention, and were unsuccessfully relied upon as anticipating it.

It is true that this construction of the Dental Vulcanite Patent narrows the scope of the patent. It is urged, with much force, that if this be the true construction, it would follow that if an inventor invented at the same time a new process and a new product, he would by such a construction of his patent lose the benefit of it, unless the infringer used his process or an equivalent one to produce his prodnct or an equivalent one. One answer to this objection is, that in the case supposed, the inventor might patent both the new process and the new product, and thus fully protect himself. In its application to this case, it is believed that the objection is without force, for the reason that such a construction of the claim of this patent is the only one which makes the claim a valid claim. To abandon the construction which makes the product patented the new manufacture, when made by the described process, is to abandon that which gives it its vitality. It is better to adopt that construction, which, although limiting the scope of the claim, secures to the inventor all that he actually invented and no more than to adopt one which would render the patent invalid, or one which, being broader than the invention of the patentee, should be a barrier in the way of future progress in discovery and invention.

Bill dismissed.

